# Cohort Profile of the Japan Collaborative Cohort Study at Final Follow-up

**DOI:** 10.2188/jea.JE20120161

**Published:** 2013-05-05

**Authors:** Akiko Tamakoshi, Kotaro Ozasa, Yoshihisa Fujino, Koji Suzuki, Kiyomi Sakata, Mitsuru Mori, Shogo Kikuchi, Hiroyasu Iso

**Affiliations:** 1 Hokkaido University Graduate School of Medicine, Sapporo, Japan; 2 Radiation Effects Research Foundation, Hiroshima, Japan; 3 University of Occupational and Environmental Health, Kitakyushu, Fukuoka, Japan; 4 Fujita Health University School of Health Sciences, Toyoake, Aichi, Japan; 5 Iwate Medical University, Morioka, Japan; 6 Sapporo Medical University School of Medicine, Sapporo, Japan; 7 Aichi Medical University School of Medicine, Nagakute, Aichi, Japan; 8 Osaka University Graduate School of Medicine, Osaka, Japan

**Keywords:** JACC Study, cohort study, Japan, follow-up

## Abstract

The Japan Collaborative Cohort Study for Evaluation of Cancer Risk (JACC Study) was established in the late 1980s to evaluate the risk impact of lifestyle factors and levels of serum components on human health. During the 20-year follow-up period, the results of the study have been published in almost 200 original articles in peer-reviewed English-language journals. However, continued follow-up of the study subjects became difficult because of the retirements of principal researchers, city mergers throughout Japan in the year 2000, and reduced funding. Thus, we decided to terminate the JACC Study follow-up at the end of 2009. As a final point of interest, we reviewed the population registry information of survivors. A total of 207 (0.19%) subjects were ineligible, leaving 110 585 eligible participants (46 395 men and 64 190 women). Moreover, errors in coding date of birth and sex were found in 356 (0.32%) and 59 (0.05%) cases, respectively, during routine follow-up and final review. Although such errors were unexpected, their impact is believed to be negligible because of the small numbers relative to the large total study population. Here, we describe the final cohort profile at the end of the JACC Study along with selected characteristics of the participants and their status at the final follow-up. Although follow-up of the JACC Study participants is finished, we will continue to analyze and publish study results.

## INTRODUCTION

To evaluate the risk impact of lifestyle factors and levels of serum components on human health, in the late 1980s we established a large-scale cohort study, the Japan Collaborative Cohort Study for Evaluation of Cancer Risk (JACC Study). During a follow-up period of approximately 20 years, data on deaths from major causes such as stomach cancer, lung cancer, and cardiovascular diseases enabled examination of risk factors. We subsequently published results regarding associations between lifestyle factors and health status in almost 200 original research articles in peer-reviewed English-language journals. Additionally, we are currently developing a website to increase public awareness.^1^

The enthusiasm of researchers is always important in promoting a cohort study, but enthusiasm is not enough since such work takes many years to bear fruit. A substantial budget is also required. The JACC Study was started after receiving a promise of funds for 10 years; however, after the initial 10 years had passed, it became necessary to apply for small public grants to maintain and follow cohort participants. In addition, administrative mergers of cities, towns, and villages throughout Japan in the year 2000 sometimes caused further difficulties in following subjects in the study area, due to changes in partnerships between local governmental offices and researchers. Moreover, with the retirement of key researchers, it was not always easy to transfer their work to their successors. As a result of these challenges, we decided to terminate follow-up of participants in the JACC Study at the end of 2009.

As a final point of interest, we used population registers in the study area to review the list of survivors. Some subjects were found to be no longer living in the study area, although the overall number of such participants was small. Moreover, a small number of errors in the coding of date of birth and sex were identified during follow-up data collection. Here we describe the final cohort profile obtained upon completion of the JACC Study. Data on cancer incidence have not yet been compiled because of the time lag of the cancer registry system. This process is expected to continue until 2013, at which point incidence information until 2009 will be made available.

## METHODS

### Study subjects

Details of the study design and concept have been described elsewhere.^2^^–^^4^ Briefly, the JACC Study was a multicenter collaborative study in which 24 institutions voluntarily participated. Recruitment of study subjects living in 45 areas was managed by individual investigators whose responsibility was to construct the cohort in that area. Data were collected from 1988 through 1990. However, although most baseline surveys were performed during this 3-year period, some subjects were recruited before and after this period because of the need for a preliminary study in 3 areas and later collaboration in 1 area. Individual informed consent before participation in the study was obtained in 36 of the 45 study areas (written consent in 35 areas and oral consent in 1 area); in the remaining 9 areas, group consent from the area leader was obtained. Participant eligibility was verified by individual investigators, who confirmed that (1) the participant was living within the study area and (2) was aged 40 to 79 years at baseline. In addition, date of birth and sex were further verified using official documents and/or a completed self-administered questionnaire.

### Follow-up

As follow-up information, dates and causes of death were annually or biannually confirmed, with the permission of the Director-General of the Prime Minister’s Office (Ministry of Public Management, Home Affairs, Post and Telecommunications) and/or the Ministry of Health, Labor and Welfare, Japan. The date of move-out of cohort members from the study area was also annually or biannually verified by the investigator in cooperation with key members of the local governmental office. In 24 of the 45 areas, data on cancer incidence such as date of diagnosis and primary site were also collected through population-based cancer registers or by reviewing the records of local major hospitals. In most areas, follow-up was completed at the end of 2009; however, it was stopped at the end of 1999 in 4 areas, at the end of 2003 in another 4 areas, and at the end of 2008 in 2 areas.

### Final data setup: correction of birth date and sex information, identification of decedents and subjects who had moved, and deletion of ineligible participants

To confirm if study participants had survived and were living in the study area at the end of follow-up, we conducted a systematic review of population registers of cohort members in 17 areas followed until 2009. In the remaining 18 areas followed until 2009, annual or biannual follow-up surveys were routinely performed using population registers; thus, no further reviews were conducted. If data from participants presumed to survive were found to be missing at the end of 2009, attempts were made to obtain information on their mortality status or current location, and relevant information was added to the follow-up data. A few participants were found to have never lived in the study area and were thus excluded from the baseline data.

This review process revealed some errors in coding of date of birth and sex. Moreover, during the merge of follow-up data with baseline identifiable data (name, date of birth, and sex), further errors in date of birth and sex were found. All such errors were corrected.

## RESULTS

Of 110 792 participants aged 40 to 79 years at baseline, 207 (0.19%) were found to have never lived in the study area. As a result, 110 585 participants (46 395 men and 64 190 women) were ultimately deemed eligible as subjects for the JACC Study, with 707 136 and 1 025 703 person-years of follow-up for men and women, respectively. Errors in the coding of date of birth and sex were found in 356 (0.32%) and 59 (0.05%) cases, respectively, during routine follow-up and final review. Table 1 shows the age and sex distribution of study participants. There were no subjects from the Shikoku region. As compared with the overall distribution of the Japanese population in 1989, our cohort participants were slightly older and included a higher percentage of women.

**Table 1.** Age distribution of cohort members at baseline by region

		Age at baseline								Total	%

		40–44	45–49	50–54	55–59	60–64	65–69	70–74	75–79		
Men											
	Japan general population 1989 (×1000)	5022	4562	3967	3706	3122	2049	1507	1169	25 104	
		20.0	18.2	15.8	14.8	12.4	8.2	6.0	4.7	100.0	
	
	JACC Study participants	5991	5794	6309	7690	8415	5516	4021	2659	46 395	100.0
	%	12.9	12.5	13.6	16.6	18.1	11.9	8.7	5.7	100.0	
	
	Hokkaido	191	182	211	267	284	201	86	43	1465	3.2
	Tohoku	809	625	797	1050	1270	894	494	293	6232	13.4
	Kanto	1325	1231	1219	1320	1446	1115	707	447	8810	19.0
	Chubu	1736	1646	1560	1763	1804	1167	916	691	11 283	24.3
	Kinki	960	908	1148	1456	1419	996	651	459	7997	17.2
	Chugoku	220	374	452	886	1251	589	770	509	5051	10.9
	Kyushu	750	828	922	948	941	554	397	217	5557	12.0

Women											
	Japan general population 1989 (×1000)	4989	4613	4052	3852	3426	2825	2141	1770	27 668	
		18.0	16.7	14.6	13.9	12.4	10.2	7.7	6.4	100.0	
	
	JACC Study participants	7536	7912	9088	10 792	11 102	8589	5548	3623	64 190	100.0
	%	11.7	12.3	14.2	16.8	17.3	13.4	8.6	5.6	100.0	
	
	Hokkaido	310	310	433	436	382	257	93	37	2258	3.5
	Tohoku	959	963	1412	1670	1670	1136	604	372	8786	13.7
	Kanto	1428	1438	1442	1605	1744	1577	892	542	10 668	16.6
	Chubu	1872	1669	1833	1933	2107	1613	1225	882	13 134	20.5
	Kinki	1253	1219	1508	1784	1566	1300	876	623	10 129	15.8
	Chugoku	300	796	828	1479	2194	1795	1289	844	9525	14.8
	Kyushu	1414	1517	1632	1885	1439	911	569	323	9690	15.1

Table 2 shows the follow-up results, and Table 3 shows the major causes of death up to 2009. These values include the follow-up information (death or move-out from the study area) that was reported in 10 of 17 areas for 516 subjects (0.5%) through a systematic review of population registers of cohort members. Finally, 27 410 deaths (24.8%; 15 401 men, 12 009 women) and 6402 move-outs (5.8%; 2343 men, 4059 women) were identified during the median 18.0-year follow-up. The first cause of death was cancer among men (37.6%) and circulatory disease among women (33.7%), and the second cause of death was circulatory disease (27.8%) and cancer (30.8%), respectively (Table 3). Among those who died of cancer, the first, second, and third leading causes of death were cancer of the lung (23.2%), stomach (18.4%), and liver (10.7%) among men and cancer of the stomach (15.4%), lung (11.2%), liver, and pancreas (9.2% for both) among women. When cancers of the colon and rectum were grouped together, that category was the second leading cause of death (12.7%) among women.

**Table 2.** Follow-up status until 2009 by sex and age

	Age at baseline								Total

	40–44	45–49	50–54	55–59	60–64	65–69	70–74	75–79	
Men									
No. at baseline	5991	5794	6309	7690	8415	5516	4021	2659	46 395
No. of deaths	394	658	1113	2000	3252	3056	2782	2146	15 401
%	6.6	11.4	17.6	26.0	38.6	55.4	69.2	80.7	33.2
No. who left study area	539	377	303	298	292	242	180	112	2343
%	9.0	6.5	4.8	3.9	3.5	4.4	4.5	4.2	5.1
Person-years	107 048	102 338	108 465	124 421	123 896	74 267	43 689	23 012	707 136
Mortality rate (per 1000 person-years)	3.7	6.4	10.3	16.1	26.2	41.1	63.7	93.3	21.8
Women									
No. at baseline	7536	7912	9088	10 792	11 102	8589	5548	3623	64 190
No. of deaths	242	368	637	1218	1982	2544	2632	2386	12 009
%	3.2	4.7	7.0	11.3	17.9	29.6	47.4	65.9	18.7
No. who left study area	605	488	479	522	606	592	483	284	4059
%	8.0	6.2	5.3	4.8	5.5	6.9	8.7	7.8	6.3
Person-years	134 927	139 091	159 465	182 347	174 721	125 510	71 076	38 566	1 025 703
Mortality rate (per 1000 person-years)	1.8	2.6	4.0	6.7	11.3	20.3	37.0	61.9	11.7

**Table 3.** Mortality distribution according to cause of death during entire follow-up period

Cause of death			Men											Women										
	
			Age at baseline								Total	%	%^a^	Age at baseline								Total	%	%^a^
	
			40–44	45–49	50–54	55–59	60–64	65–69	70–74	75–79				40–44	45–49	50–54	55–59	60–64	65–69	70–74	75–79			
All causes			394	658	1113	2000	3252	3056	2782	2146	15 401	100.0		242	368	637	1218	1982	2544	2632	2386	12 009	100.0	

A00–B99	Certain infectious and parasitic diseases		6	10	18	38	56	62	44	33	267	1.7		4	4	18	31	62	50	43	36	248	2.1	

C00–D49	Neoplasms		160	312	542	927	1425	1073	792	561	5792	37.6	100.0	147	182	319	563	740	714	618	414	3697	30.8	100.0
C15		Esophagus	12	14	28	42	55	38	17	10	216		3.7	0	1	5	3	4	7	8	7	35		0.9
C16		Stomach	32	62	87	176	252	199	151	109	1068		18.4	19	26	33	93	91	127	104	76	569		15.4
C18		Colon	12	14	36	41	67	59	44	35	308		5.3	2	13	29	45	62	65	65	52	333		9.0
C19–C20		Rectum	8	17	26	52	39	30	27	22	221		3.8	9	8	12	18	35	16	26	11	135		3.7
C22		Liver and intrahepatic bile ducts	21	46	79	128	167	77	66	37	621		10.7	8	12	29	65	81	77	33	35	340		9.2
C23		Gall bladder	1	5	6	16	17	32	12	12	101		1.7	4	7	11	15	17	28	35	13	130		3.5
C24		Other and unspecified parts of biliary tract	5	11	11	34	41	42	28	16	188		3.2	3	8	11	22	31	37	37	23	172		4.7
C25		Pancreas	13	20	29	50	78	63	43	42	338		5.8	7	16	26	48	82	66	62	33	340		9.2
C33–C34		Lung	27	50	114	205	364	290	181	114	1345		23.2	18	20	39	54	96	78	70	40	415		11.2
C50		Breast	0	1	0	0	0	0	1	0	2		0.0	28	26	28	37	29	18	17	9	192		5.2
C53		Cervic uteri												6	2	10	5	9	5	7	5	49		1.3
C54		Corpus uteri												2	2	7	7	9	3	4	2	36		1.0
C55		Uterus, part unspecified												2	3	1	3	13	9	8	7	46		1.2
C56		Ovary												13	8	15	16	22	10	9	5	98		2.7
C61		Prostate	2	4	20	21	68	49	59	56	279		4.8											
C64		Kidney	0	4	7	12	14	9	12	4	62		1.1	0	0	1	5	3	11	5	1	26		0.7
C65–C67		Urothelial tract	2	7	13	11	40	31	34	17	155		2.7	1	0	6	6	21	14	16	14	78		2.1
C82–C85		Non-Hodgkin’s lymphoma	0	8	17	29	44	20	15	15	148		2.6	5	6	10	25	23	17	12	7	105		2.8
C90		Multiple myeloma	2	7	4	12	18	12	9	5	69		1.2	4	4	9	12	15	15	11	10	80		2.2
C92		Myeloid leukemia	5	10	11	16	17	7	9	3	78		1.3	1	4	4	12	15	9	8	3	56		1.5

E00–E89	Endocrine, nutritional and metabolic diseases		8	10	17	29	38	35	27	28	192	1.2		2	4	7	10	36	49	48	43	199	1.7	

G00–G99	Diseases of the nervous system		4	7	17	19	50	39	18	10	164	1.1		1	4	12	23	44	27	29	13	153	1.3	

I00–I99	Diseases of the circulatory system		86	132	252	460	857	908	919	673	4287	27.8		52	70	138	306	585	913	1001	978	4043	33.7	
I20–I25		Ischemic heart disease	34	45	69	124	199	204	181	147	1003			11	8	34	51	105	188	176	185	758		
I48		Atrial fibrillation and flutter	0	0	4	10	19	25	24	15	97			1	0	1	3	16	21	29	26	97		
I50		Heart failure	7	19	26	56	121	151	178	153	711			8	5	22	44	101	180	200	239	799		
I60–I69		Cerebrovascular disease	30	44	113	194	362	389	408	285	1825			24	43	63	130	256	393	461	407	1777		
I71		Aortic aneurysm and dissection	4	4	12	21	44	40	38	15	178			2	3	2	17	22	29	28	13	116		

J00–J99	Diseases of the respiratory system		14	40	62	219	408	501	550	500	2294	14.9		3	18	23	67	182	281	357	354	1285	10.7	
J09–J18		Influenza and pneumonia	6	20	30	115	228	273	327	327	1326			2	11	15	39	110	173	247	245	842		
J43		Emphysema	0	1	6	19	58	58	64	44	250			0	0	0	2	2	4	4	4	16		

K00–K95	Diseases of the digestive system		28	35	53	78	82	109	80	46	511	3.3		1	12	13	54	54	106	91	82	413	3.4	
K74		Fibrosis and cirrhosis of liver	16	16	27	34	20	13	19	6	151			1	8	6	23	22	31	19	10	120		

N00–N99	Diseases of the genitourinary system		2	9	14	33	67	68	67	59	319	2.1		2	3	15	22	51	78	82	81	334	2.8	
N17–N19		Acute kidney failure and chronic kidney disease	2	7	12	22	50	52	52	53	250			1	2	12	17	38	50	63	60	243		

R00–R99	Symptoms, signs, and abnormal clinical and laboratory findings, not elsewhere classified		4	4	6	7	26	52	99	109	307	2.0		1	1	1	7	26	84	172	234	526	4.4	
R54		Age-related physical debility	0	0	0	4	19	37	87	99	246			0	0	1	2	18	71	150	224	466		

S00–T88	External causes		78	86	113	150	170	150	126	93	966	6.3		22	57	72	97	143	147	117	73	728	6.1	

	Others		4	13	19	40	73	59	60	34	302	2.0		7	13	19	38	59	95	74	78	383	3.2	

^a^Percentage of deaths per neoplasm.

## DISCUSSION

This final profile of the JACC Study Group describes the number of participants and their follow-up status. During the median 18-year follow-up, we found errors in the coding of date of birth and sex data as well as incorrectly registered cases. Accordingly, we would advise future researchers planning a field study to thoroughly check participant eligibility and basic information such as date of birth and sex; this can be performed at least twice, by using a population register and a self-questionnaire.

Although follow-up information was annually or biannually confirmed, 516 subjects who had died or moved out of the study area were not identified during routine follow-up. The use of population registers to verify that subjects are living in the study area is therefore necessary because it enables identification of deceased individuals and those who have moved out of the study area. Furthermore, 356 (0.32%) and 59 (0.05%) cases of incorrect coding of date of birth and sex, respectively, were found during routine follow-up and final review. Miscoding of data can occur by verification only once, and miscoding of date of birth and sex information may cause errors such as merging of the follow-up information of 1 participant with the baseline data of another participant. Thus, careful efforts such as independent double-entry are essential to reduce such miscoding.

The JACC Study is one of the largest cohort studies in Japan. Selected characteristics of study participants were similar to those of the Japanese general population, and thus, the JACC Study can be regarded as representative of the Japanese population, though it should be noted that no subjects were recruited from the Shikoku region. Almost 200 original articles on the risk factors for cancer, cardiovascular disease, and other diseases have been published using the results of the JACC Study. It was not an easy task to establish and maintain such a large collaborative cohort study with a limited budget; the voluntary efforts of the collaborators were essential. Although unexpected errors were found, we believe that the impact of these errors was negligible because the number of ineligible cases and amount of missing data were small relative to the large total study population.

Cohort studies need to continue over a long period if they are to yield fruitful results. Moreover, because all study participants must be followed up carefully and thoroughly, considerable funding is required. The JACC Study received systematic support for the first 10 years, at which point this funding ceased and maintenance and follow-up of cohort participants was accomplished by means of smaller grants. The retirements of principal researchers and city mergers throughout Japan made it difficult to continue follow-up. Thus, we decided to terminate the follow-up of participants in the JACC Study at the end of 2009. Our experience indicates that the development and maintenance of an appropriate long-term management system is essential when launching a cohort study and that adequate and steady support from funding bodies is also important.

We would like to express our sincere thanks to all participants and researchers related to the JACC Study, and to all the funding bodies that supported our study. Hereafter, we plan to use the final dataset and remaining sera to examine the risk impact of lifestyle factors and levels of serum components on human health.

## References

[1] The JACC Study Group. JACC Study. [cited 2012 October 3]. Available from: http://publichealth.med.hokudai.ac.jp/jacc/

[2] Ohno Y, Tamakoshi A; JACC Study Group Japan collaborative cohort study for evaluation of cancer risk sponsored by Monbusho (JACC Study). J Epidemiol. 2001;11:144–50 10.2188/jea.11.144 11512570 PMC11735075

[3] Tamakoshi A; Japan Collaborative Cohort Study for Evaluation of Cancer Overview of the Japan Collaborative Cohort Study for Evaluation of Cancer (JACC). Asian Pac J Cancer Prev. 2007;8 Suppl:1–8 18260701

[4] Tamakoshi A, Yoshimura T, Inaba Y, Ito Y, Watanabe Y, Fukuda K, Profile of the JACC Study. J Epidemiol. 2005;15 Suppl 1:S4–8 10.2188/jea.15.S4 15881191 PMC8565873

